# Jacov Tal (1940 – 2005): remembrances of a friend

**DOI:** 10.1186/1742-4690-2-12

**Published:** 2005-02-22

**Authors:** Gabriel Kaufmann, Kuan-Teh Jeang

**Affiliations:** 1 Department of Biochemistry, Tel Aviv University, Tel Aviv 69978, Israel; 2 Laboratory of Molecular Microbiology, NIAID, NIH Bethesda, Maryland 20892, USA

## Abstract

An obituary commemorates the life and works of Jacov Tal.

A friend's passing elicits a set of emotions. Collecting and sharing remembrances are steps of closure in bidding farewell. Here, we honor and remember Jacov Tal (Fig. 1) who passed away on February 8^th^, 2005. At the time of his passing, Jacov was the Head of Virology at Ben-Gurion University Medical School, Israel. In brief, four of us, who befriended Jacov in different capacities, write our remembrances. It is appropriate to recall the words of a colleague who on another occasion upon the passing of a giant in American science remarked, "Well, ghosts can't make men do anything!" Thus, the true reflection of a person is not what (s)he through his/her prestige, wealth, and position makes others do in life, but how (s)he is remembered by others in death. Jacov started as a young retrovirologist in J. M. Bishop and H. Varmus' laboratory; and it is fitting that he is remembered by friends in *Retrovirology*.

**Figure 1:**
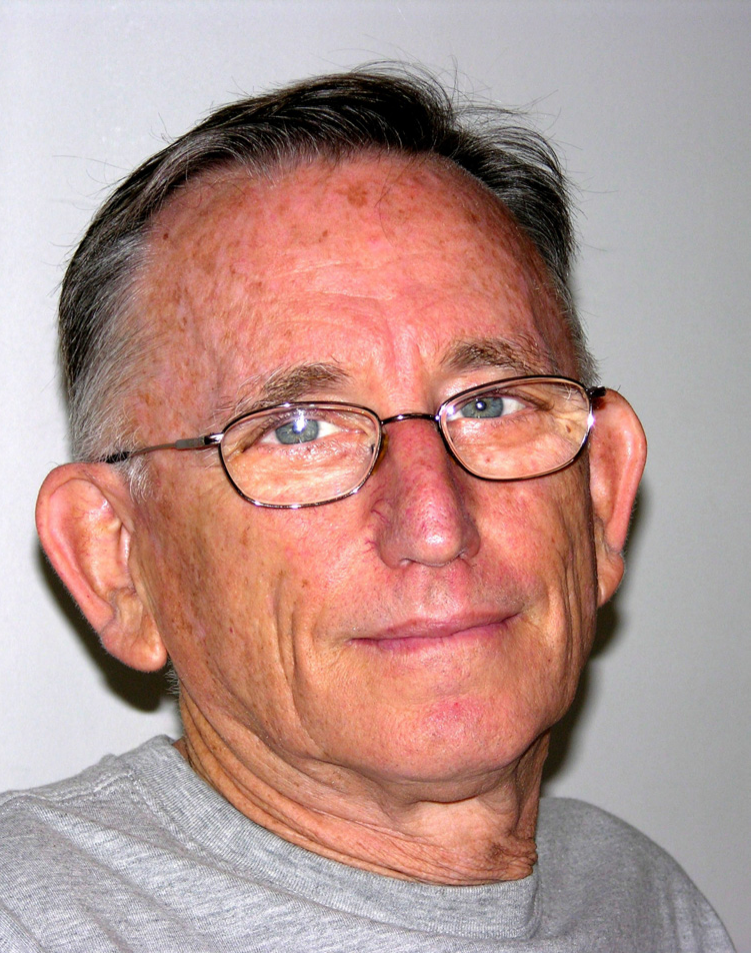
Jacov Tal, circa 2004.

"I remember Jacov as my actual mentor in my first year as a graduate student in the laboratory of Uri Littauer that Jacov had joined a year earlier. Jacov took similar care of other new comers interested in nucleic acids and molecular biology including Hiroshi Inouye, Inder Verma and Jacque Beckman, even to the point of neglecting his own work. Ibelieve that Jacov's selfless involvement in public affairs and later decision to become a virologist date back to a childhood experience; namely, only a ruling elite and diplomatic corpse in a country that Jacov's parents were stationed in, knew about the polio epidemic, considered then an ideological insult.

Jacov Tal studied at the Hebrew University in Jerusalem where he obtained the Bachelor degree in Biochemistry and Microbiology in 1964 and the Master degree in Biochemistry in 1966. He continued his studies at the Weizmann Institute of Science where he obtained his PhD degree in Biochemistry in 1971. Jacov Tal did his postdoctoral training first with Hershel Raskas in Washington University where he investigated adenovirus gene expression and later with Harold Varmus and Michael Bishop at USCF where he studied the relation between the retroviral genome and the genome of its cellular host. After these formative years as a molecular virologist Jacov Tal joined the newly founded Ben Gurion University in the Israeli desert city Beer Sheva where he initiated and led the Medical School's Virology Department." (Gabriel Kaufmann)

"Professor Jacov Tal will be best remembered by the scientific community for his extensive studies of the parvoviruses adeno-associated virus (AAV) and minute virus of mice (MVM). One of his significant achievements was the determination of parameters of site-specific integration of AAV, leading to development of potential vectors for gene therapy. Other important contributions were his insights into MVM's ability to kill cancerous cells, while leaving normal cells unaffected. The students of Professor Tal will also remember him for his dedication to training them to be rigorous and discerning scientists, and his concern for their well being. His colleagues will miss his sharp wit and analytic acumen." (Maureen Friedman)

"Jacov Tal and I collaborated closely to his last days. Together, we found that during embryonic development MVM somehow senses a differentiation signal, and we suggested a relation between this observation and the MVM's anti tumor cell activity. I recall Jacov's 'freshman' enthusiasm in private, and his public posture as one who speaks up his mind without hesitation; standing up against any perceived injustice." (Claytus Davis)

"I met Jacov towards the latter years of his career. In 1993, Jacov came to the US to do a sabbatical in Peter Chiang's laboratory. By and by, he drifted into my laboratory and actually spent the entire year working with me. Jacov by then, already a senior scientist for many years, not unexpectedly struggled heroically (and largely unsuccessfully) at the bench; and certainly it was not his bench-skills that impressed me. What did impress me was Jacov's common sense and his very human and generous attitudes. I recall an incident during Jacov's first week when he had not yet gotten to know all the members of my lab. At that time, there was a tall, curly-haired, darkly-handsome and academically gifted young man, working as a post-doc with me, who had graduated from Yale, obtained his MD degree from Duke, and received house staff and infectious diseases training from the University of Virginia. This person also has four siblings who are MDs. Jacov upon meeting this young man, whispered to me excitedly, 'Now here is a nice Jewish boy who is going to make the mother of a Jewish daughter very happy!' Surprise, surprise...that person turned out not to be Jewish, but a Catholic Lebanese-American of Arabic descent. Afterwards, a sheepish Jacov explained to me that it is very difficult to nearly impossible to tell between an Arab and a Jew in Israel; and as far as he was concerned, it made no difference whether Arab-American or Jewish-American. I was struck by his frankness and openness. In his typically thoughtful and 'dovish' ways, over the next many years, Jacov would email me from Israel his periodic 'roadmaps' for peace in the Middle East, accompanied by his incisive commentaries. I will deeply miss my friend's common sense advice and humor." (Kuan-Teh Jeang)

